# An Experimental Murine Model to Study Acquisition Dynamics of Tick-Borne Langat Virus in *Ixodes scapularis*

**DOI:** 10.3389/fmicb.2022.849313

**Published:** 2022-04-14

**Authors:** Waqas Ahmed, Kundave V. Rajendran, Girish Neelakanta, Hameeda Sultana

**Affiliations:** Department of Biomedical and Diagnostic Sciences, College of Veterinary Medicine, University of Tennessee, Knoxville, TN, United States

**Keywords:** ticks, mice, Langat virus, acquisition, transstadial transmission, blood feeding, *Is*SMase expression

## Abstract

*Ixodes scapularis* ticks acquire several pathogens from reservoir animals and transmit them to humans. Development of an animal model to study acquisition/transmission dynamics of these pathogens into and from ticks, respectively, is challenging due to the fact that in nature ticks feed for a longer duration and on multiple vertebrate hosts. To understand the complex nature of pathogen acquisition/transmission, it is essential to set up a successful tick blood feeding method on a suitable vertebrate host. In this study, we provide evidence that murine model can be successfully used to study acquisition dynamics of Langat virus (LGTV), a member of tick-borne flaviviruses. Mice were inoculated intraperitoneally with LGTV that showed detectable viral loads in blood, skin, and other tissues including the brain. Both larval and nymphal ticks that were allowed to feed on the murine host successfully acquired LGTV loads. Also, we found that after molting, LGTV was transstadially transmitted from larval to nymphal stage. In addition, we noted that LGTV down-regulated *Is*SMase expression in all groups of ticks possibly for its survival in its vector host. Taken together, we provide evidence for the use of murine model to not only study acquisition dynamics of LGTV but also to study changes in tick gene expression during acquisition of arboviruses into ticks.

## Graphical Abstract

Tick-borne virus acquisition study model.

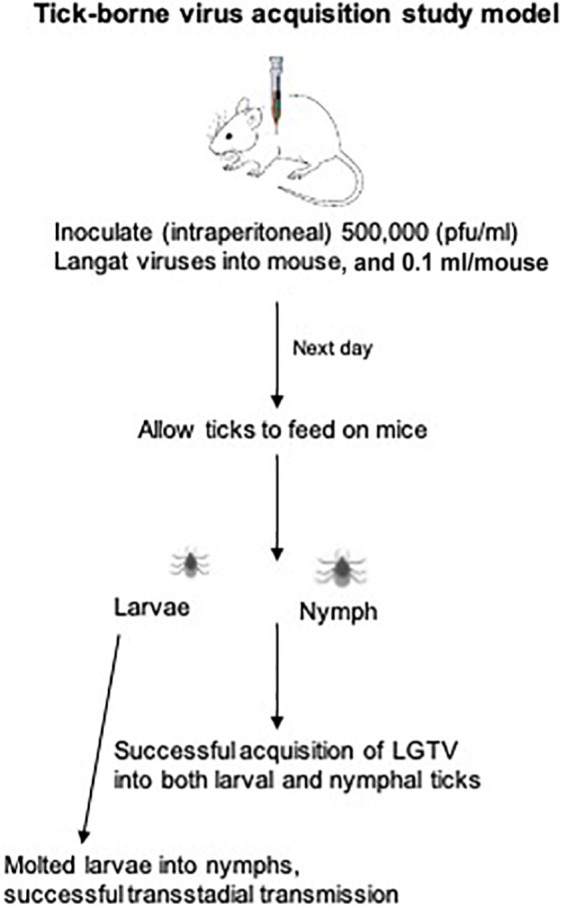

## Introduction

Tick-borne flaviviruses are recognized as one of the major global concerns to human health. These viruses are mostly transmitted through the bite of an infected *Ixodes* tick that results in meningitis, encephalitis, biphasic fever, febrile illness, or hemorrhagic fever in humans (Kellman et al., 2018; Madison-Antenucci et al., 2020). Tick-borne flaviviruses including Tick-borne encephalitis virus (TBEV), Powassan virus (POWV), Langat virus (LGTV), Kyasanur forest disease virus (KFDV), Louping ill virus, and Omsk hemorrhagic fever virus (OHFV) are both genetically and antigenically related to each other (Hermance and Thangamani, 2018; Kellman et al., 2018; Hermance et al., 2019; Pierson and Diamond, 2020). Several studies, including our own, have primarily focused on studying tick-bacteria interactions (Khanal et al., 2017, 2018; Taank et al., 2018, 2020; Dahmani et al., 2020; Ramasamy et al., 2020; Regmi et al., 2020). In contrast, a limited number of studies have focused on understanding interactions between tick-borne flaviviruses and their arthropod vectors at the molecular level (Neelakanta and Sultana, 2015; Grabowski and Hill, 2017; Wolf et al., 2020). Therefore, a detailed understanding of the connection between tick-borne flaviviruses and their vector host is essential to design preventive strategies and to disrupt the pathogen transmission.

Langat virus is a naturally attenuated flavivirus with non-pathogenic/low-pathogenic concerns to human health. Therefore, our laboratory used it as a model pathogen to understand the interactions of virulent tick-borne flaviviral infections in ticks. In addition, work with LGTV can be safely performed at the Biosafety level 2 (BSL-2) (Zhou et al., 2018). Like other tick-borne flaviviruses, LGTV has a lipid-envelope and a positive-sense single-stranded RNA genome of approximately 11 kb in length (Radzi et al., 2015). Arthropods play key role(s) in the acquisition and transmission of numerous viruses in different geographic regions (Nuttall and Labuda, 2003; Shaw and Catteruccia, 2019). Ticks have developed paramount strategies to indulge as successful vectors for pathogen acquisition and transmission. They have an ability to efficiently take a blood meal through biting a host and allowing viral survival and replication within them for an extensive period of time (Nuttall et al., 2000; Mitzel et al., 2007; Hart and Thangamani, 2020; Boulanger and Wikel, 2021). Ixodid ticks are reported to be the most prominent vectors that play key role(s) in the flavivirus acquisition and transmission cycle (Mitzel et al., 2007). Flaviviruses can infect Ixodid ticks at any developmental stage (larvae, nymphs, or adults), and the infection can be maintained throughout the tick’s lifespan (Mitzel et al., 2007; Bowman and Nuttall, 2008; Hart and Thangamani, 2020). Tick-borne flaviviruses usually spend 95% of their life cycle in *Ixodes* tick vectors rather in the vertebrate host including the accidental human host (Maffioli et al., 2014).

Regardless of the significance of Ixodid ticks as key vectors for bacterial acquisition and transmission, little is known regarding the flavivirus-vector host interactions. Laboratory models mimicking natural routes of infection of animals and ticks with tick-borne viruses are urgently needed to study molecular mechanisms of virus transmission. An understanding of the molecular mechanism(s) is essential to gain in-depth knowledge on the acquisition of the virus from the vertebrate host to ticks, pathogen replication in the tick midgut, crossing of the mid-gut barrier, persistent survival in the salivary glands, and then transmission to the vertebrate host. Technical challenges faced in a research laboratory have limited the number of studies conducted on the flavivirus-tick host interactions. The two common methods used for infecting ticks in a laboratory condition are parenteral inoculation by microinjection (Talactac et al., 2017) and synchronous infection of ticks by immersion method (Mitzel et al., 2007). There are several limitations for these two methods, as majority of the flaviviruses need high biocontainment level facilities (such as BSL-3 or BSL-4), which add to the technical issues on working with these pathogens. Microinjection method to generate LGTV-infected ticks is an artificial system. The success in generating LGTV-infected ticks by microinjection is facilitated by the evasion of the midgut escape barrier to allow direct access into the hemolymph. This process also ensures a relatively quicker progression into the tick-salivary gland, however, generating cohorts of infected ticks with equal pathogen burden is a major limitation. This factor is a critical determinant to assess vector competency in these artificially infected ticks (Talactac et al., 2017; Sun et al., 2020). Tick salivary glands enhance virus replication that can be detected in females at least upon 120 days post-infection (Nuttall and Labuda, 2003; Slovák et al., 2014). For synchronous LGTV-infection of ticks, large volumes (1–2 ml) of concentrated virus stocks (such as 10^7^ or 10^8^ pfu/ml) and longer incubations (17 days) are required for generation of small batch of infected ticks. Also, only 60–70% of ticks were capable of acquiring pathogen loads by body absorbance or bathing in the virus stocks (Taank et al., 2018; Regmi et al., 2020).

Our previous studies have demonstrated the role of arthropod-derived extracellular vesicles (EVs or exosomes) in the transmission of flaviviruses such as LGTV, and DENV2/3, which perhaps are mediated by the CD63 ortholog Tsp29Fb (Vora et al., 2018; Zhou et al., 2018, 2020). *I. scapularis* ISE6 cells have shown that LGTV might utilize the tick-derived molecules to facilitate its replication in the host cells (Grabowski et al., 2016, 2017). In addition, we noted that LGTV infection suppress *Ixodes scapularis Is*SMase gene expression, a molecule involved in exosome biogenesis (Regmi et al., 2020). *Is*SMase (Sphingomyelinase D or SMase D, a venomous protein ortholog of spiders) is a tick saliva component that regulates the cytokine expression to modulate the programming of vertebrate immune response via arthropod exosomes (Wikel, 2017; Regmi et al., 2020). *Is*SMase neutralizes the Th1 cytokine response toward a Th2-induced cytokine response and modulates CD4 + T cells in order to express interleukin 4 (IL-4) (Wikel, 2017; Regmi et al., 2020). Our recent studies focusing on understanding the molecular mechanisms during pathogen-vector-host interactions have revealed different survival strategies used by microbes (Taank et al., 2018, 2020; Vora et al., 2018; Zhou et al., 2018, 2020; Dahmani et al., 2020; Ramasamy et al., 2020; Regmi et al., 2020). Until this study, evaluating vector competence of *I. scapularis* for LGTV upon blood feeding has never been investigated. In the current study, utilizing LGTV as a model pathogen, we established a method to infect naïve *I. scapularis* by feeding these ticks on LGTV-infected blood from murine host. This study not only gives a novel method to generate LGTV-infected *I. scapularis* ticks but also provides an efficient tool to study acquisition dynamics of this important vector.

## Materials and Methods

### Mice and Ticks

**FIGURE 1:**
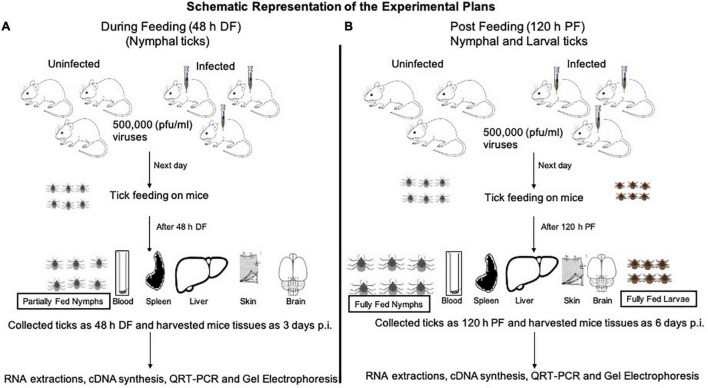
Experimental design of LGTV infection in mice and ticks. Mice were injected intraperitoneally (i.p.) with diluted virus containing a dose of 5 × 10^4^ (50,000 pfu/mouse followed by nymph or larval tick attachment and feeding. Uninfected nymphal or larval unfed ticks were allowed to attach and feed on uninfected or LGTV-infected mice. Three mice were used in each group (uninfected or LGTV-infected groups). **(A)** Partially fed nymphal ticks (48 h during feeding; DF group) were pulled off the body of mice. Mice were euthanized and blood and tissues (Sp, spleen; Lv, liver; Sk, skin; Br, brain) were harvested at day 3 post-infection (p.i.). **(B)** Nymphal or larval ticks were allowed to fully engorge and repleted ticks were collected as 120 h post-feeding/PF group. To generate the PF group of nymphal or larval ticks, we used two independent batches (one batch for nymphal and other batch for larval tick feeding) of uninfected or LGTV-infected mice (three mice in each group), respectively. Mice were euthanized at day 6 p.i. and blood and tissues (Sp, Lv, Sk, and Br) were harvested. Mice blood, tissues, and ticks were processed for RNA extractions, cDNA synthesis, followed by QRT-PCR analysis and DNA agarose gel electrophoresis.

Laboratory reared *I. scapularis* ticks (larvae and nymphs) were obtained from a continuously maintained colony from BEI resources/Center for Disease Control and Prevention (CDC) and used in this entire study. C57BL/6 mice (females, 6 weeks old, Charles River Laboratories, United States) were used in this study. All experiments were performed in strict accordance with the recommendations in the “Guide for Care and Use of Laboratory Animals of the NIH, United States.” Mice studies were performed based on animal protocol approved by the Institutional Animal Care and Use Committee (IACUC). Animal husbandry and administration of tranquilizer during animal experiments was performed as reported previously (Taank et al., 2018; Regmi et al., 2020). The schematics show the experimental design for virus acquisition by ticks (nymphal or larval acquisition of LGTV loads) carried out in this study (Figures 1A,B).

### Langat Virus Infection and Replication in Mice

For the tick feeding/virus acquisition experiment, a total of twelve mice were clustered into four groups. Three mice were considered for each of the four groups and are as follows: (1) uninfected group of mice (that allow generation of partially fed uninfected ticks; these ticks were pulled off from mice and referred in this study as 48 h DF ticks), (2) uninfected group of mice (that allow generation of fully engorged or repleted uninfected group of ticks; these ticks were collected after repletion and referred in this study as 120 h PF ticks), (3) LGTV-infected mice (that were used to collect infected-DF ticks), and (4) LGTV-infected mice (that were used to collect infected-PF ticks, as above). For the larval tick feeding/acquisition experiment, six mice were grouped into two sub-groups (uninfected or LGTV-infected), with three mice in each sub-group. Ticks were collected at only one time point as post-feeding-PF group.

Wild type LGTV (LGT-TP21 Strain, obtained from BEI Resources) is used in this study. Virus stocks were made in Vero cells and titers were determined in same cells by end-point virus dilution assays and as described in our published study (Zhou et al., 2018). Virus dilution was prepared from the laboratory virus stocks of 1 × 10^9^ plaques forming units (pfu/ml). Mice in the LGTV-infected group were injected intraperitoneally with 0.1 ml (50,000 pfu/mouse) of diluted virus suspension in 1X PBS containing 1% gelatin (SIGMA Aldrich). After 3 or 6 days p.i. with LGTV, mice were euthanized at the given time point of tick collection as (DF)- or (PF)-groups. Murine blood and tissues (such as spleen, liver, skin, and brain) were harvested for downstream processing such as RNA isolation, cDNA synthesis, and Quantitative Real-Time PCR (QRT-PCR) analysis to detect viral loads.

### Feeding of Nymphal/Larval Ticks on Langat Virus-Infected Mice

Naïve nymphal/larval ticks were fed on uninfected or LGTV-infected mice (injected intraperitoneally with 50,000 pfu/mouse) to demonstrate the virus acquisition from the murine host into the tick body. Tick infestations were initiated upon 1 day post infection of mice. Briefly, ticks were placed near the neck region of the mouse and they were allowed to freely move and chose their attachment site (that appeared mostly to be behind the ear regions). Mice were individually housed in constrained cages with a metal grid (∼1 inch above the bottom of the cage) and moist paper towels were kept below the grid to collect repleted ticks. Nymphal ticks were fed on mice and collected at two given time points of 48 h DF (partially fed ticks) or 120 h PF (fully engorged or repleted ticks), for further analysis. These time points of tick collection also correspond to day 3 or 6-post LGTV-infection of mice. Partially fed nymphal ticks attached to the body of the mice were pulled off with forceps during blood feeding (and these ticks are referred as 48 h DF ticks). Fully fed/engorged nymphal/larval ticks (that completed blood meal are referred as 120 h PF ticks) were collected continuously (as repleted group and on daily basis) from the uninfected or LGTV-infected mice. Uninfected nymphal/larval ticks fed (partially or fully, respectively) on naïve/uninfected C57BL/6 mice were used as control for both DF and PF groups of ticks. Fully engorged nymphal/larval ticks were continuously collected after repletion (or dropped off the mice) from 3 to 5 days of infestation. After nymphal/larval tick repletion, mice were euthanized and blood/tissues (such as spleen, liver, skin, and brain) were harvested at day 6 p.i. of mice and processed for RNA isolation, cDNA synthesis, and QRT-PCR analysis to detect LGTV loads. Independently, a large batch of larval ticks (∼80–100 of uninfected or LGTV-infected ticks) were fed on uninfected or LGTV-infected mice (as described previously) and these fed larvae were allowed to molt into nymphal stage for 4–6 weeks. To molt larval ticks, larvae were placed in a humidity-controlled incubator (Parameters Generations and Control, NC, United States) at 23 ± 2°C with 95% relative humidity and a 14/10-h light/dark photoperiod regiment. Molted nymphs were collected and stored at −80°C until needed for further analysis.

### Isolation of Total RNA, cDNA Synthesis, and Quantitative Real-Time PCR Analysis in Mice Tissues and Ticks

Total RNA was generated from naïve or LGTV-infected mice blood, harvested tissues (such as spleen, liver, skin, and brain), partially fed (48 h DF), or fully fed nymphal or larval ticks (120 h PF) or molted nymphs by using the Aurum Total RNA Mini kit (Bio-Rad, United States) and following the manufacturer’s instructions. During RNA extractions, on-column DNaseI digestion was performed as per the manufacturer’s recommendations. The eluted RNA was converted to cDNA using a cDNA synthesis kit (Bio-Rad, United States). The generated cDNA was used as a template for the QRT-PCR reactions to analyze the viral loads or the *Is*SMase transcript levels. QRT-PCR was performed using CFX96 or CFX-Opus QRT-PCR system (Bio-Rad, United States), and iQ-SYBR Green Supermix (Bio-Rad, United States), and the isolated cDNA samples. In QRT-PCR reactions, the quantity of mice or tick *beta-actin* transcripts are used to normalize the amount of template in each reaction. To determine viral loads, levels of LGTV transcript were quantified in the cDNA samples. The standard curve for each gene fragment was generated using 10-fold serial dilutions starting from 1 to 10^–5^ ng of known quantities of respective fragments. For standard preparation, initial DNA concentration was measured by taking optical density readings using a TECAN plate reader (TECAN, United States). After measurement of concentrations, 10-fold serial dilutions were made to prepare various standards. Oligonucleotides for mice/tick actin used in QRT-PCR analysis are published in our previous studies (Taank et al., 2018; Zhou et al., 2018). In addition, oligonucleotides used to detect LGTV loads and *Is*SMase transcript levels are also published in our previous studies (Zhou et al., 2018; Regmi et al., 2020). After completion of QRT-PCR cycles, products were analyzed on 1.2–1.5% agarose gels containing ethidium bromide. Internal quality control included parallel PCR amplifications of no template control (NTC) and positive control (sequenced standard fragments). LGTV prM-E, *Is*SMase, mice, and tick beta actin PCR amplified products were gel purified and confirmed by sequencing for primer specificity. Sequence confirmed PCR products were serially diluted and used as standard that serve as positive control for agarose gel electrophoresis. QRT-PCR products obtained from LGTV specific oligonucleotides from every experimental group including murine blood, tissues, fed nymphs (DF and PF), and larvae (PF) (from both acquisition experiments) were gel purified and sequenced at the Eurofins Genomics facility (United States).

### Statistics

Statistical significance in the data sets was analyzed using GraphPad Prism6 software and Microsoft Excel 2010. For data to compare two means, the non-paired student’s *t*-test was performed. *P*-values of <0.05 were considered significant in all analyses. Statistical tests and *P*-values used in the study are shown in data sets.

## Results

### Langat Virus Infection Is Detected in Murine Blood and Other Tissues

**FIGURE 2:**
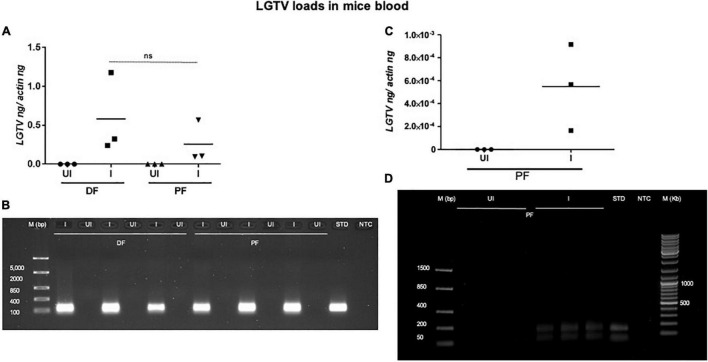
Detection of LGTV loads in blood from mice used for nymphal/larval tick feeding. **(A)** QRT-PCR analysis showing LGTV loads in mice blood that were used for nymphal tick feeding. Levels of LGTV transcript was normalized to mice beta-actin levels. **(B)** Amplification of LGTV fragment with expected band (143 bp) is shown using agarose gel electrophoresis image in samples generated from mice blood infected with LGTV. Standard used in the QRT-PCR analysis serves as a positive control on gel. NTC is no template control, and M represents marker in bp. ns indicates no significance between the days 3 or 6 p.i. of mice. QRT-PCR analysis showing LGTV loads in blood from mice that were used for larval tick acquisition of the pathogen. **(C)** LGTV transcript levels were normalized to mice beta-actin levels. **(D)** Amplification of LGTV fragment with expected band (143 bp) is shown using agarose gel electrophoresis in samples generated from mice blood infected with LGTV. Standard used in the QRT-PCR serves as a positive control on gel. NTC indicates no template control and M represents marker in bp or Kb.

**FIGURE 3:**
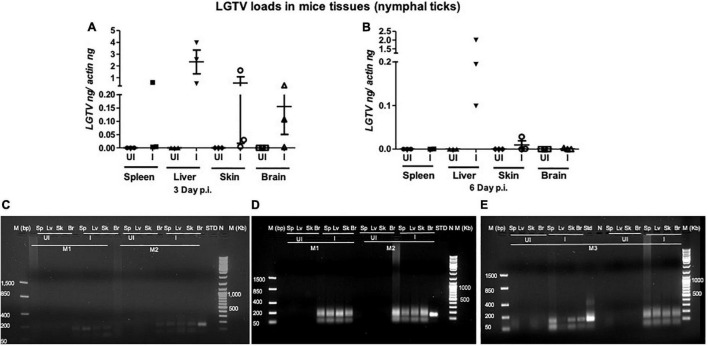
LGTV disseminated from blood and replicated in mice tissues. QRT-PCR analysis showing LGTV loads in cDNA samples extracted from mice spleen (Sp), liver (Lv), skin (Sk), and brain (Br) tissues collected at either day 3 p.i. **(A)** or at day 6 p.i. **(B)**. LGTV transcript levels were normalized to mice beta-actin levels. (**C–E**, mouse 1–3). Agarose gel electrophoresis image showing amplification of LGTV (143 bp) in mice tissues harvested at day 3 p.i. (group of mice used to generate 48 h DF ticks) or at 6 days p.i., (group of mice used to generate 120 h PF ticks). Standard 4 **(C)** or 3 **(D)** or 2 **(E)** was used in the QRT-PCR reactions as positive controls. NTC indicates no template control and M represents marker in either bp or Kb.

**FIGURE 4:**
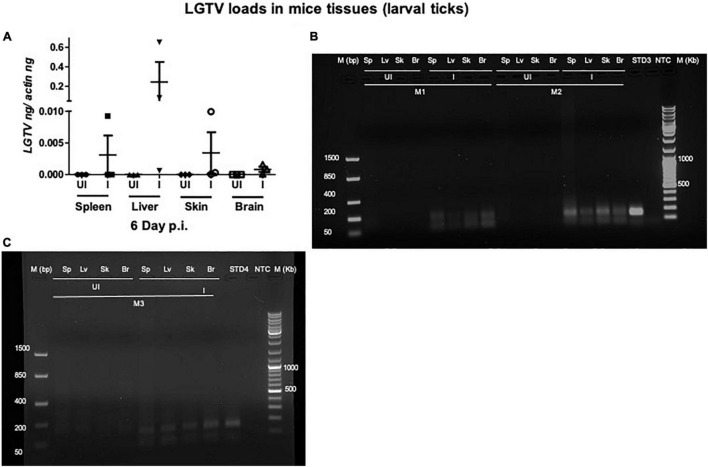
Detection of LGTV loads in tissues from mice used for larval tick feeding. **(A)** QRT-PCR analysis showing LGTV loads in cDNA samples from mice tissues such as spleen (Sp), liver (Lv), skin (Sk), and brain (Br) collected at 6 days p.i. LGTV transcript levels were normalized to mice beta-actin levels. Agarose gel electrophoresis images showing amplification of LGTV levels (143 bp) in mice tissues at 6 days p.i. Data from mice 1 and 2 (M1 and M2) is shown in panel **(B)** and data from mouse 3 (M3) is shown in panel **(C)**. Standard 3 or 4 used in QRT-PCR represents positive control on gels. NTC indicates no template control and M represents marker in bp and Kb.

This study was conducted to demonstrate the ability of *I. scapularis* ticks to acquire LGTV loads from the infected mice. Therefore, we determined viral loads in murine blood and other tissues (such as liver, spleen, skin, and brain). At day 3 and 6 p.i. of mice, LGTV loads were highly detectable in blood samples of all mice. QRT-PCR analysis showed that LGTV readily infected mice with increased viral loads in blood detected at an early stage of infection (3 days p.i.) in comparison to the later stage of infection (6 days p.i.) (Figure 2A). QRT-PCR amplification showed LGTV (143 bp) product on 1.5% agarose gel electrophoresis in both the infected groups of mice (3 or 6 days, p.i.), while the uninfected control group showed no amplification of products (Figure 2B). Another subsequent experiment with larval ticks also showed successful LGTV infection in mice via needle inoculation (Figure 2C). The gel images showed amplicons of LGTV product (143 bp) in the infected group, while no product was detected in the uninfected mice (Figure 2D). At 3 days p.i., LGTV loads were detected to be higher in all tested tissue samples, compared to the levels noted at day 6 p.i. (Figures 3A,B). At 6 days p.i., the tissues remained positive for LGTV infection with detectable viral loads. Among the tested tissues and at both time points of LGTV-infection, higher detectable loads were noted in liver, followed by skin, spleen, and brain (Figures 3A,B). The agarose gel images confirmed the presence of amplicons (product of 143 bp) in the LGTV-infected mice tissues (Figures 3C–E). Tissues were also collected from the mice that were used for larval tick feeding. LGTV loads in these mice showed similar trend as noted in samples generated from mice used for nymphal ticks (Figures 3B, 4A). All mice tissues remained positive for LGTV loads in these group of mice that allowed larval tick feeding. Liver showed higher viral loads followed by skin, spleen, and brain (Figure 4A), a trend similar to samples generated from mice used for nymphal tick feeding. Gel electrophoresis analysis confirmed the presence of LGTV amplicons in all infected mice tissues (Figures 4B,C). These results indicated a successful infection of mice with detectable LGTV loads in mice blood and tissues.

### Acquisition of Langat Virus Loads by Nymphal/Larval Ticks

**FIGURE 5:**
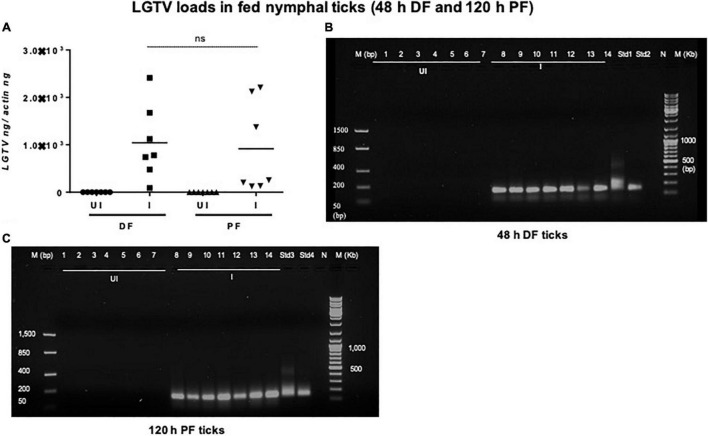
Detection of LGTV loads in partially or fully fed nymphal ticks. **(A)** QRT-PCR analysis showing LGTV loads in cDNA samples of nymphal ticks collected from mice at 3 days p.i., (48 h during feeding, DF or partially fed ticks) or at 6 days p.i., (120 h feeding, PF ticks). LGTV transcript levels were normalized to tick beta-actin levels. Agarose gel electrophoresis images shows amplification of LGTV (a product of 143 bp) in nymphal ticks collected at either (DF) **(B)** or (PF) time points **(C)**. Standards 1 and 2 **(B)** or 3 and 4 **(C)** used in QRT-PCR served as positive controls on gels. NTC indicates no template control and M represents marker in bp and Kb.

**FIGURE 6:**
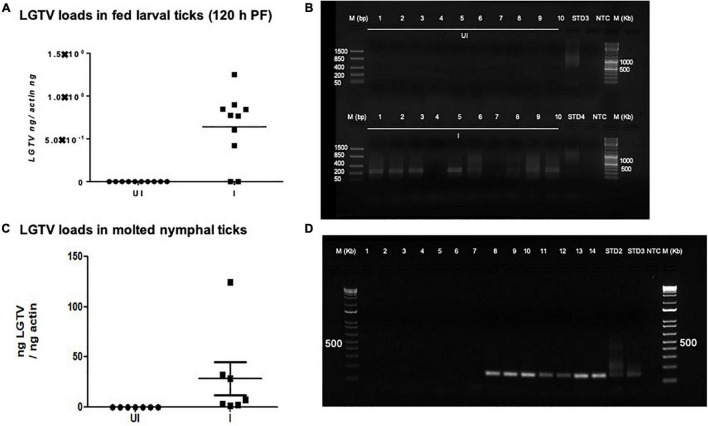
Detection of LGTV loads in larval ticks and molted nymphs. **(A)** QRT-PCR analysis showing LGTV loads in cDNA samples of larval ticks collected at 6 days p.i., of mice (120 h PF ticks). **(B)** Agarose gel electrophoresis image showing amplification of LGTV (143 bp) in larval ticks collected at 120 hpost-feeding. QRT-PCR analysis **(C)** or PCR amplification **(D)** is shown to reveal the LGTV levels in molted nymphs. Levels of LGTV transcripts were normalized to tick beta-actin levels. Standards 3 and 4 **(B)** or 2 and 3 **(D)** used in QRT-PCR were used as positive controls on gels. NTC indicates no template control and M represents marker in bp and Kb.

Viral loads were highly detectable in all tested ticks that included both the partially fed group (of 48 h DF) or the fully fed/engorged repleted group of ticks (120 h PF) (Figure 5A). No significant difference in viral loads were noted between ticks collected during 48 h DF or 120 h PF groups (Figure 5A). Amplification of LGTV product (143 bp) was recorded as an enhanced signal in both DF and PF group of ticks (Figures 5B,C). Larval ticks were allowed to feed completely on LGTV-infected or uninfected control mice. These ticks were collected after repletion and were considered as 120 h PF larval group. QRT-PCR analysis revealed the presence of viral loads in individually processed larval ticks that were fully fed to repletion (Figure 6A). Agarose gel electrophoresis of QRT-PCR products confirmed the presence of amplicons corresponding to 143 bp of LGTV fragment (Figure 6B). Except for two fed larval ticks, all other ticks showed the presence of amplicons and were positive for LGTV loads (Figures 6A,B). These results indicate that naïve nymphs/larvae that fed on LGTV-infected mice are capable of acquiring the virus from the murine host.

### Detection of Transtadial Transmission of Langat Virus From Infected Larval Ticks to Molted Nymphs

Fully fed larvae (120 h PF) repleted from LGTV-infected or uninfected control group of mice were collected and allowed to molt into nymphs. QRT-PCR analysis revealed the presence of viral loads in all individually molted nymphs (Figure 6C). PCR products run on agarose gel electrophoresis also confirmed the presence of amplicons corresponding to LGTV fragment of 143 bp (Figure 6D). These results showed that LGTV acquisition from murine host by larval ticks allows transstadial transmission of the pathogen to the molted nymphs.

### *Is*SMase Expression Is Reduced in Ticks That Acquired Langat Virus Loads From Mice

**FIGURE 7:**
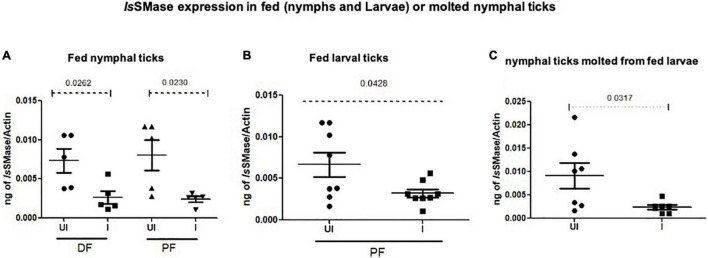
*Is*SMase expression is downregulated upon LGTV acquisition and in molted nymphs. QRT-PCR analysis showing *Is*SMase gene expression levels in ticks collected during (48 h DF) or post-feeding (120 h PF) and freshly molted nymphs. *Is*SMase transcript levels are shown in nymphal ticks **(A)** (collected from 48 h DF or 120 h PF) or in larval ticks **(B)** (collected from 120 h PF ticks) or in molted nymphs **(C)**. *Is*SMase mRNA levels were normalized to tick beta-actin levels. *P-*value determined by Student’s two-tailed *t*-test is shown.

To support the use of this murine model to study changes in the arthropod gene expression during pathogen acquisition into ticks, we determined the *Is*SMase transcript levels in partially or fully fed nymphal/larval ticks and in freshly molted nymphs. QRT-PCR analysis showed that *Is*SMase transcript levels were significantly reduced in LGTV-infected nymphal ticks that were collected either during feeding (48 h DF) or post-feeding (120 h PF) in comparison to the levels noted in the respective group of ticks fed on the uninfected control group of mice (Figure 7A). Also, *Is*SMase levels were significantly downregulated in larvae (120 h PF) that fed on LGTV-infected mice when compared to the levels noted in larvae fed on uninfected mice (Figure 7B). In addition, *Is*SMase transcript levels in molted nymphs were significantly reduced in LGTV-infected larval ticks when compared to the nymphs that fed on the uninfected control group of mice (Figure 7C). Collectively, these data suggests that LGTV downregulates *Is*SMase in both partially or fully fed nymphal/larval ticks during its acquisition from murine host or in molted nymphs that acquired the pathogen loads by transstadial transmission.

## Discussion

The developmental cycle of an arthropod vector from an egg through larva and/or nymph to the adult stage could take years (Mitzel et al., 2007; Stewart and Bloom, 2020). Arthropod vectors such as *I. scapularis* ticks feed on the vertebrate host for about 3–6 days to acquire a complete blood meal (or full engorgement) and this extended event allows successful acquisition and/or transmission of pathogens (Heinze et al., 2012; McNally et al., 2012; Stewart and Bloom, 2020). The ability of ticks to transstadially transmit flaviviruses provide greater possibilities for the evolutionary changes in the virus at the phenotype and/or genotype level in the vector (Kellman et al., 2018). Consequently, thoughtful investigations to study the interactions between an arthropod vector and the tick-borne flaviviruses are essential to understand an in-depth mechanism of viral pathogenesis.

To combat vector-borne viral diseases, novel methods need to be developed to address prevention and control strategies. Development of simple approaches to infect ticks would rapidly advance investigations in learning vector-host interactions. Several studies have reported the acquisition and subsequent transmission of bacterial pathogens from the vertebrate host to ticks such as *I. scapularis*, the vector of *Borrelia burgdorferi* (Levin and Fish, 2000; Bockenstedt et al., 2006), human granulocytic ehrlichiosis (Hodzic et al., 1998), and transmission of *Anaplasma phagocytophilum* (Levin and Ross, 2004). Michael Levin and colleagues infected the mice with *A. phagocytophilum*, and upon feeding of larval ticks they demonstrated the pathogen loads in ticks. The infected larval ticks were allowed to molt, and the nymphs were found to have maintained the bacterial loads (Levin and Ross, 2004). So far, we believe that no acquisition studies have been recorded and shown for tick-borne viruses such as LGTV. Our current study provides evidence for the acquisition of tick-borne LGTV by both nymphal and larval ticks upon feeding on infected mice. In this study, we fed the nymphal and larval ticks on LGTV-infected mice and observed that ticks acquired a significant level of viral burden that was detectable in both nymphal (partially or fully fed) and larval ticks. Furthermore, arthropod vector such as, *Amblyomma tigrinum*, *A. ovale*, and *A. tonelliae* larvae effectively acquired the flaviviruses after feeding on viremic chicks (Flores et al., 2019). The technical difficulties of the previously used methods for infecting ticks, such as parenteral inoculation (Talactac et al., 2017) and synchronous infection of ticks by immersion method, restrict key studies of the tick-virus interactions at the higher containment levels (Mitzel et al., 2007). Our previous studies (Taank et al., 2018; Regmi et al., 2020) have shown that synchronous infection of ticks with LGTV or *A. phagocytophilum* is possible in 60–70% of ticks; however, it would take 17 days of incubation. Also, the immersion method is arduous to assess with high priority pathogens such as POWV and TBEV due to the requirement of higher containments/facilities and the use of larger volumes of concentrated viruses (at the doses of 10^7^–10^9^ pfu/ml or perhaps much higher dose).

Studies that determine tropism of LGTV in rodents are limited. In the current study, we reported a simple method of acquisition of LGTV infection by a large number of larval or nymphal ticks feeding on murine blood. Mice administered intraperitoneally with 5 × 10^4^ pfu of LGTV dose showed replicative viral loads on day 3 p.i., however, the viral infection persisted until day 6 p.i., in blood and peripheral tissues such as spleen, liver, and skin (Figure 2). Also, we found that LGTV disseminated to the mice brain tissue, suggesting compromised blood-brain barrier (BBB) and neuroinvasion of the virus. This data suggested that inoculation with viral dose of 5 × 10^4^ pfu is required for LGTV replication and dissemination into mice tissues. Also, detection of viral loads in mice blood and skin indicated the LGTV spread in the murine host. The detection of higher viral loads in the peripheral tissues (such as spleen and liver) suggested enhanced replication at these sites. The dissemination of viral loads into the brain further suggests that high viremia in blood and peripheral tissues could lead to the breach of the BBB and neuroinvasion. In addition, detection of LGTV in partially fed nymphal ticks also demonstrated higher viral loads suggesting an early and successful acquisition of virus into ticks. The detection of LGTV loads at 120 h PF (or day 6 p.i. for mice) of nymphal or larval ticks further suggested successful acquisition, replication, and persistence of virus in blood-fed ticks collected at later time point of mice infection. Transstadial transmission of LGTV from infected larval ticks to molted nymphs further suggested that acquisition of pathogen loads by larval ticks is transmitted to the next tick stage and is perhaps maintained in the tick body. Our recent study showed that LGTV suppress *Is*SMase transcripts for its survival and replication in unfed and partially fed (24 h DF) nymphal ticks (Regmi et al., 2020). Down-regulation of *Is*SMase expression in LGTV-infected nymphs (partially or fully fed), larvae, and molted nymphs suggested the pathogen influence of gene expression in all stages of ticks. This study has also shown that *Is*SMase reduced levels correlates with down regulation of its enzymatic activity and accumulation of sphingomyelin (SM) lipid levels that may support membrane associated viral replication and exosome biogenesis upon LGTV infection in tick cells. We report an appropriate, fast, and efficient method to generate LGTV infected blood fed ticks (as large batches). Compared to the other approaches such as microinjections or synchronous infections, we believe that this method is a natural way to generate large batches of LGTV-infected ticks.

In summary, viral loads were detected both in murine tissues and in nymphal/larval ticks fed on LGTV-infected mice suggesting viral-replication in mice blood and dissemination into tissues including skin and, furthermore, acquisition into the nymphal/larval tick’s body from the infected murine host. We have also provided evidence for the transstadial transmission of LGTV from fed larval ticks to molted nymphs. Our study provides a method not only to generate LGTV-infected ticks but also provides a tool to study acquisition and transmission dynamics of this virus and perhaps other high priority tick-borne flaviviruses, such as TBEV and POWV. This study would considerably boost investigation, not only in understanding the viral acquisition and transmission and possibly other vector-host interactions, but could also accelerate anti-vector/transmission-blocking vaccine research in the field of tick-borne viral diseases.

## Data Availability Statement

The original contributions presented in the study are included in the article, further inquiries can be directed to the corresponding author.

## Ethics Statement

The animal study was reviewed and approved by University of Tennessee, College of Veterinary Medicine, Institutional Animal Care and Use Committee (IACUC).

## Author Contributions

GN infected mice and generated fed *I. scapularis* uninfected or LGTV-infected ticks. WA and KR performed all molecular analysis and wrote the first manuscript draft. HS modified, revised, and finalized the manuscript, collected all required materials and reagents, designed and coordinated the entire study, organized all the data, and compiled and supervised overall investigations. All authors performed experiments, discussed, analyzed, and interpreted the data in several settings, read and edited the manuscript, and approved the submitted version.

## Conflict of Interest

The authors declare that the research was conducted in the absence of any commercial or financial relationships that could be construed as a potential conflict of interest.

## Publisher’s Note

All claims expressed in this article are solely those of the authors and do not necessarily represent those of their affiliated organizations, or those of the publisher, the editors and the reviewers. Any product that may be evaluated in this article, or claim that may be made by its manufacturer, is not guaranteed or endorsed by the publisher.
